# Robust phylogenetic profile clustering for *Saccharomyces cerevisiae* proteins

**DOI:** 10.7717/peerj.19370

**Published:** 2025-04-28

**Authors:** Paul M. Harrison

**Affiliations:** Department of Biology, McGill University, Montreal, Quebec, Canada

**Keywords:** Gene origins, Phylogenetic profiles, Clustering, Intrinsic disorder, Prion-like, Protein function

## Abstract

**Background:**

Genes are continually formed and lost as a genome evolves. However, new genes may tend to appear during specific evolutionary epochs rather than others, or disappear together in a more recent organismal clade. Methods to identify gene origination might simply use the last common ancestor to contain an ortholog as the putative gene origination point, or use a heuristic threshold that allows for a certain amount of missing orthologs in the cohort of species examined. Here, to avoid such issues, an alternative approach based on the clustering of phylogenetic profiles is applied, and the results are examined for any evidence of epochal trends in gene origination, and associated trends in specific sequence traits or functional associations.

**Methods:**

A phylogenetic profile is simply an array indicating the presence or absence of a gene in a list of species. These profiles were compared and clustered to discern patterns in gene occurrences across >800 fungal species, centering the analysis on the budding yeast *Saccharomyces cerevisiae*.

**Results:**

Clear epochs of gene origination were observed linked to the last common ancestors of *Saccharomycetaceae* and *Saccharomycetes*, and also to *Fungi* and earlier ancestors. These trends are independent of the proteome and genome-assembly quality of the underlying data. Clusters of phylogenetic profiles demonstrated some significant functional associations, such as to cellular spore formation and chromosome segregation in genes originating in *Saccharomycetaceae*. The phylogenetic profile clustering analysis enabled detection of parameter-independent trends in intrinsic disorder, prion-like composition and gene uniqueness as a function of epochal gene age. For example: new proteins with prion-like domains have arisen at a similar rate for most of fungal evolution centred on *S. cerevisiae*; the most proteins with mild intrinsic disorder have appeared during the early *Saccharomycetaceae* epoch rather than more recently, and very recently formed genes are the least likely to be single-copy (*i.e*., ‘unique’ yeast proteins).

**Conclusions:**

For individual proteins, the profile cluster data generated here are useful for investigating experimental hypotheses, since they provide evidence for functional linkages that have yet to be discerned.

## Introduction

New genes arise continually during evolution. Such genes can then be deeply conserved or lost sporadically. Concerted appearance of new genes may occur in defined phylostrata, *i.e*., at nodes in the tree of life. Efforts to probe such strata have been dubbed phylostratigraphy (Domazet-Loso, Brajkovic & Tautz, 2007).

Phylostrata are typically assigned based on the most divergent species containing an ortholog (Carvunis et al., 2012; Montanes et al., 2023; Roginski et al., 2024; Vakirlis et al., 2024). Some studies augment this with synteny information for closely-related species, *e.g*., within the *Saccharomyces* genus (Vakirlis et al., 2024). Barrera-Redondo et al. (2023) analyzed the number of taxonomic levels extending to the most divergent ortholog and allowed an absence of ortholog detection for up to 70% of these levels. Thus, estimated origination time of genes can be reliant on initial taxonomic filters with variable or arbitrary thresholds.

Here, to address the problem of defining phylostrata, with the yeast *Saccharomyces cerevisiae* as an example, a data-driven approach based on the concept of phylogenetic profiles is applied to yield an alternative perspective (Pellegrini, 2012; Pellegrini et al., 1999). Patterns of gene origination can be discerned by analyzing phylogenetic profiles which are arrays indicating the presence or absence of corresponding orthologs across interrogated proteomes. For genes originating in the same epochs and experiencing similar clade-specific patterns of gene loss, such profiles should be similar enough to cluster together, and also robust to sporadic errors in ortholog detection that deform a phylogenetic profile at one or a handful of positions. Because of large-scale genome sequencing efforts, we now have a resource of several hundred fungal proteomes from high-quality genome assemblies (Grigoriev et al., 2014; Hittinger et al., 2015). Thus, clustering of such lengthy phylogenetic profiles may be informative about gene origination trends for the yeast *S. cerevisiae* and indicate how the signal for such trends might or might not be obscured by proteome quality, which is a direct consequence of genome assembly quality and annotation quality. Lower-quality genome assemblies may have missing or fragmentary genes. Thus, they may yield errors in ortholog assignment. Indeed, the presence or absence of highly-conserved ortholog families in a proteome is a standard criterion for inferring sufficient genome assembly quality, such as in the BUSCO tool (Manni et al., 2021). Here, because clusters can be assessed using varying thresholds for cluster coherence, we have surveyed for parameter-independent trends in sequence features such as intrinsic disorder, prion-like composition and gene uniqueness (*i.e*., lack of paralogs). Furthermore, significant functional associations for profile clusters have been searched for and discerned.

## Methods

### Proteome data

The UniProt fungal reference proteomes (totalling >800) were downloaded from www.uniprot.org in January 2024 (File S1). The *Saccharomyces cerevisiae* proteome used as the focus of this analysis is strain 288c (UniProt proteome identifier UP000000625).

### Proteome quality

BUSCO (Benchmarking Universal Single-Copy Ortholog) and CPD (Complete Proteome Detector) scores were used to assess proteome quality (Manni et al., 2021; The UniProt Consortium, 2023). BUSCO is a tool to judge the completeness of a genome assembly (and consequently of an annotated proteome), by checking for the presence of deeply-conserved, single-copy genes across an organismal clade (Manni et al., 2021). CPD is a filter used by the UniProt database to statistically assess the completeness and quality of a proteome by comparing it to a group of closely-related species, and classifying proteomes as *Standard*, *Close to Standard* or *Outlier* based their protein count relative to what is expected within a specific taxonomic clade (The UniProt Consortium, 2023). To assess the effect of proteome quality on the results below, three different proteome lists are drawn up. Proteomes are included in the ‘*HQ2*’ list if their BUSCO C score is ≥97%, or their CPD score is *Standard* or *Close to Standard (High Value)*. They are included in the ‘*HQ1*’ list if that have BUSCO C score ≥95%, or CPD *Standard* or *Close to Standard* or *Outlier (High Value)* (File S1). Any other proteomes are labelled ‘*all’* (for the set of all the proteomes).

### Orthologs

Sets of orthologs to each yeast (UP000000625) protein were calculated as described previously (Su & Harrison, 2020), using the bi-directional best hits method and a BLASTP e-value threshold of 1e−03 (Altschul et al., 1997).

### Profile calculation

For each set of orthologs, an evolutionary profile is calculated which is a series of 1s and 0s, with a ‘1’ representing presence of an ortholog in a proteome and a ‘0’ for absence. The proteome order in the profiles is: *Saccharomyces* (genus), then other *Saccharomycetaceae* (family), then other *Saccharomycetes* (class), then other *Ascomycota* (phylum), then other *Fungi* (kingdom). Within these five ranges, the ordering is arbitrary.

### Profile clustering, multiple profile alignment & consensus derivation

The distance between any two profiles is simply the number of mismatches (*i.e*., 1 aligning to 0, or *vice versa*). This is denoted PM (from *P*rofile *M*ismatches). Sometimes this is calculated as a proportion (*i.e*., divided by profile length). Profiles are clustered to identify proteins that have similar patterns of origination and clade-specific loss. These clusters are derived using the neighbour-joining algorithm, as implemented in PHYLIP (https://phylipweb.github.io/phylip/), (Saitou & Nei, 1987). For each cluster, a multiple profile alignment is derived. This is analogous to the concept of multiple sequence alignments, with one profile for each *S. cerevisiae* protein. For each multiple profile alignment, a consensus profile is calculated, wherein the dominant character 1 or 0 is selected for each position. Then, the *consensus agreement* (abbreviated CA) is the average PM of each profile in the cluster when compared to the consensus profile.

### Evolutionary epochs and random profiles

Ideal profiles were derived for the evolutionary epochs: *S. cerevisiae*, *Saccharomycetaceae, WGD*, *Saccharomycetes, Ascomycota*, and *Fungi*. The label *WGD* refers to the organisms that have the whole-genome duplication that occurred during *Sacccharomycetes* evolution (Wolfe & Shields, 1997). These ideal profiles contain 1s for every species within the clade in question, and 0s for every other species. Consensus distance (CD) is the PM value between a cluster consensus sequence and any of these ideal profiles.

To check that trends are not biased by comparisons to the ideal profiles described above, PM values between observed profiles and two randomly generated profiles were examined. The first such profile was generated by consulting a sequence of pseudo-random numbers, with a 0.25 chance of 1 (0.75 chance of 0), and with these chance thresholds transposed for the second random profile.

### Removing cluster redundancy and profile assignment to closest epochs

CD values to the ideal profiles were used to weed the profile clusters down, removing redundancy. Clusters were sorted on increasing CD to each ideal profile, and for the same CD, on decreasing cluster size. The sorted list of clusters was then scanned, and relative to any cluster higher in the list, any clusters further down the list that had any membership overlap with it were de-selected and subsequently ignored. The selected clusters were then re-examined and given a final assignment to which of the ideal profiles they were most similar. To only consider significant CD values, thresholds of CD were calculated for a Bonferroni-corrected *P*-value threshold of 8.5e−06 (=0.05/5989) from simulated populations of random profiles with the same overall proportions of 1s and 0s observed in the actual data, and any clusters with CD values below these thresholds were discarded. These thresholds were calculated for different quality data sets (*HQ1, HQ2, all*) and ideal profiles, and ranged from 0.35 to 0.44.

Profile clusters can thus be assigned to the evolutionary epoch that they are closest to. However, higher consensus agreement (CA) and lower consensus distance (CD) thresholds can be used to produce more strict assignments to each of the six epochs. Specifically, the following sets of values were assessed: [0.99, 0.98, 0.95, 0.9, 0.8] for CA, and [0, 0.005, 0.01, 0.02, 0.05, 0.1, 0.2] for CD. Also, trends in assignment to each epoch can be analyzed for the independence of results from the parameters CA and CD.

### Quality level agreement

To check for variance in the assignment of proteins to different evolutionary epochs, the agreement in assignment to the ideal profiles between the three different quality levels (*HQ1, HQ2, all*) was calculated. This is simply the proportion of proteins in a cluster whose profiles are assigned significantly to the same closest ideal profile. This is termed quality level agreement (QLA). This was used as a measure of assignment confidence to the ideal profiles for each epoch.

### ‘Unique’ yeast proteins

If any yeast proteins had no matches in BLASTP searches of the yeast proteome against itself (with e-value thresholds of 1e−02, 1e−03 or 1e−04), they were labelled as potential ‘unique’ yeast proteins (UYPs) (Altschul et al., 1997).

### Prion-like proteins

Prions are proteins that can propagate a protein state (usually a conformation) to other copies of the same protein. Several of these have been documented in *S. cerevisiae* linked to domains that are mostly rich in glutamine and asparagine residues (An, Fitzpatrick & Harrison, 2016). Such prion-like sequence composition was assessed using the program PLAAC (Lancaster et al., 2014), with two thresholds >0.0 and ≥15.0 for the PRD score, and failing that, for the LLR score (these are two scores output by the program).

### Intrinsically-disordered proteins

Intrinsic disorder occurs when a whole protein or a protein region remains unfolded during at least part of its functioning. Intrinsic disorder was assigned with IUPRED3 (Erdos, Pajkos & Dosztanyi, 2021). Proteins were classified as ≥50% disordered, or ≥20% disordered over the whole length of their sequences.

### Gene Ontology (GO)

The Gene Ontology is a database of assignments of functional terms for known genes/proteins (The Gene Ontology Consortium et al., 2023). Significant GO term enrichments for profile clusters were examined, relative to the GO term assignments for the whole *S. cerevisiae* proteome. These were calculated using hypergeometric probability with a Bonferroni correction for the number of GO terms examined.

## Results

### Evidence for epochs of gene origination

**Figure 1:**
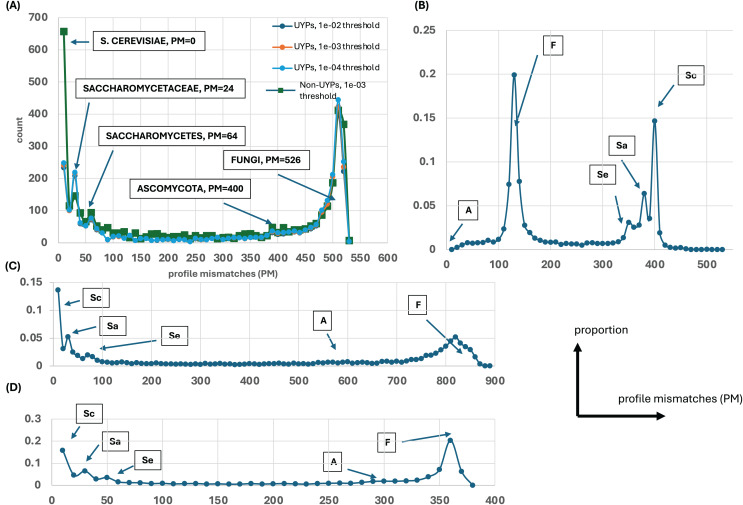
Distributions of profile mismatches (PM) yield evidence for epochs of gene origination. (A) The distribution of PM for the profiles of all yeast proteins *vs* the *S. cerevisiae* ideal profile for the *HQ1* quality level. The data is split into UYPs (unique yeast proteins) and non-UYPs for the 1e−03 BLASTP threshold of comparison of the yeast proteome against itself. Also plotted are the PM distributions for proteins labelled UYP for the 1e−02 and 1e−04 thresholds. On the plot, the ‘ideal’ values for comparison of the other ideal profiles with the *S. cerevisiae* profile are highlighted. (B) The distribution of PM for the profiles of all yeast proteins *vs* the *Ascomycota* ideal profile for the *HQ1* quality level. The ‘ideal’ PM values are also labelled here, but the labels are abbreviated as *Sc* (*S. cerevisiae*), *Sa* (*Saccharomycetaceae*), *Se* (*Saccharomycetes*), *A* (*Ascomycota*) and *F* (*Fungi*). (C) As in (A), but for the *all* quality level. Only the total distribution is shown. The ‘ideal’ PM values are also pointed out here with the abbreviated labels. (D) As in (A), but for the *HQ2* quality level. A schematic of axis labels for parts (C) and (D) is to the left of this plot.

Evolutionary profiles are a concise way to summarise the patterns of conservation of orthologs, with presence/absence of orthologs in species represented in binary strings of 1s and 0s. To discern any overall trends in gene origination during fungal evolution (relative to the budding yeast *S. cerevisiae*), these profiles for every *S. cerevisiae* protein were all compared to ideal profiles representing evolutionary epochs and the number of profile mismatches (PM) was analyzed (Fig. 1). For example, the ideal profile for the *Saccharomycetaceae* (*Sa*) epoch is a row of 1s for all the *Sa* species followed by a row of 0s for the rest of the *Fungi* (1111…11110000…0000). In addition, all the profiles were compared against two randomly-generated profiles (Fig. S1), to check whether these results arise from comparing just to the ideal profiles. Regardless of which ideal or random profile is compared against, and which level of proteome quality is considered, clear trends in the timing of gene origination are observed. Obvious peaks emerge for gene origination in *Saccharomyces*, or in the last common ancestor of *Saccharomycetaceae*, *Saccharomycetes* or in *Fungi* (or before, since for *Fungi* they may originate earlier in eukaryotic evolution). There is no obvious peak for origination after the last common ancestor of *Ascomycota* (Fig. 1).

### Basic properties of profile clusters

**Figure 2:**
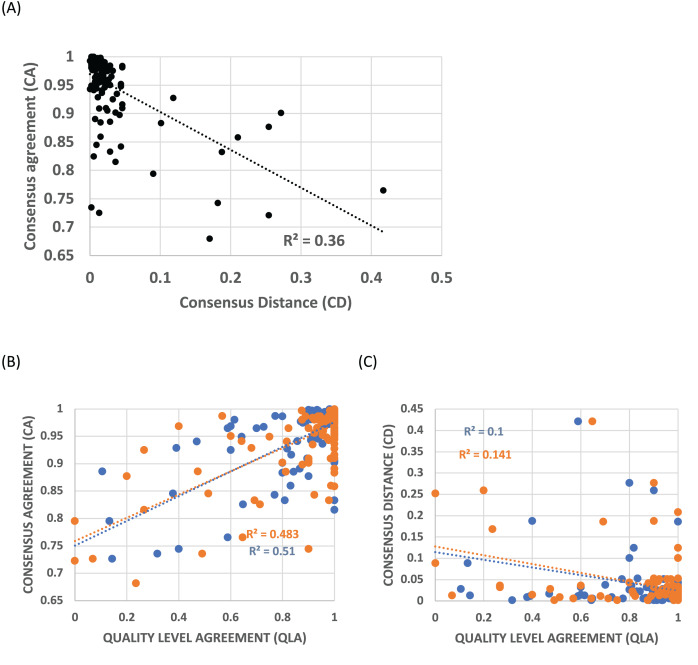
Trends in consensus agreement (CA), consensus distance (CD) and quality level agreement (QLA) for profile clusters. (A) CA *vs* CD for the *HQ1* quality level for clusters of size ≥10. Pearson correlation coefficients are labelled on the plot (*P* < 0.0001). The Spearman rank correlation coefficient for this data set is R_S_ = –0.60 (*P* < 0.0001). (B) CA for the *HQ1* quality level for clusters of size ≥10, *vs* their QLA with the *all* set (blue points), and the *HQ2* set (orange), for clusters of size ≥10. Pearson correlation coefficients are labelled on the plot (*P* < 0.0001). The Spearman rank correlation coefficients for these data sets are R_S_ ≥ 0.49 (*P* < 0.0001). (C) CD for the *HQ1* quality level for clusters of size ≥10, *vs* their QLA with the *all* set (blue points), and the *HQ2* set (orange), for clusters of size ≥10. Pearson correlation coefficients are labelled on the plot (*P* < 0.002). Spearman correlation coefficients are significant for the HQ2 data set only (*P* = 0.009); but become significant for HQ1 if the ~40% of points with QLA = 1 are removed.

Profiles were clustered as described in Methods. The cluster protein lists for the *HQ1* quality level that are the focus of this analysis are supplied in File S2, with the multiple profile alignments for each cluster in File S3. Consensus agreement (CA), a measure of the coherence of a profile cluster, is inversely correlated with the distance to the most similar epoch ideal profile that matches significantly (called consensus distance (CD), *P* < 0.0001), indicating that this cluster coherence is directly related to the occurrence of such epochs of gene origination (Fig. 2A). For the *all* and *HQ2* proteome quality levels, the Pearson R^2^ values are also very significant (0.39 and 0.43 respectively; *P* < 0.0001), and for the three sets, Spearman rank correlation coefficients have *P* < 0.0001. Profile clusters aggregate in the upper left corner of the plot, with 84% of CA values ≥0.9 and 92% of CD values ≤0.1 (Fig. 2A). Quality level agreement (QLA) is the proportion of each cluster that is also assigned to the same epoch at the other two quality levels; these confidence indicators are also strongly correlated with CA (*P* < 0.0001, Fig. 2B), but less so with CD, still significantly by Pearson correlation (*P* < 0.002), but with the HQ1 data set becoming non-significant according to Spearman rank correlation (Fig. 2C). More than three-quarters of QLA values of either sort are ≥0.9.

Table 1 lists upper bounds for numbers of genes assigned to a specific epoch of gene origination. Notably, only ~100 genes are assigned as closest to the ‘whole genome duplication’ *WGD* epoch ideal profile. Only two clusters ≥10 were assigned as closest to this *WGD* profile (these are included in the File S2 list).

**Table 1:** Upper bounds for the genes assigned to various epochs, and for different proteome quality thresholds.

Closest epoch	Quality level	Number of genes/proteins in assigned clusters
*S. cerevisiae*	All	780
	HQ1	813
	HQ2	640
*Saccharomycetaceae*	All	984
	HQ1	979
	HQ2	1,121
*WGD*	All	102
	HQ1	77
	HQ2	114
*Saccharomycetes*	All	645
	HQ1	529
	HQ2	570
*Ascomycota*	All	568
	HQ1	680
	HQ2	779
*Fungi*	All	2,657
	HQ1	2,772
	HQ2	2,740

**Figure 3:**
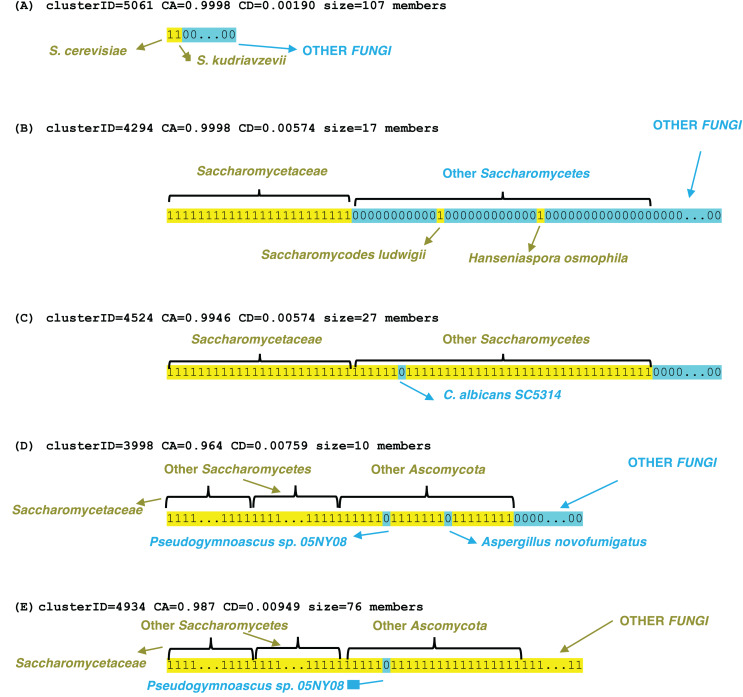
Examples of profile clusters for five different epochs of origination. They are (A) *S. cerevisiae*, (B) *Saccharomycetaceae*, (C) *Saccharomycetes*, (D) *Ascomycota*, (E) *Fungi*. The cluster ID, CD value, CA value and cluster size are listed for each. The profiles are colour-coded yellow 1 (presence of an ortholog) or blue 0 (absence). The extent of various clade levels is labelled on each example, and notable species are pointed out.

Examples of profile clusters for each of the epochs *S. cerevisiae*, *Saccharomycetaceae*, *Saccharomycetes*, *Ascomycota* and *Fungi* are illustrated (Fig. 3). Example (A) comprises a set of >100 orthologs that is observed conserved in the *Saccharomyces* species *cerevisiae* and *kudriavzevii* (File S3). The cluster in (B) is a set of 17 proteins that are conserved across *Saccharomycetaceae* and *Saccharomycodaceae*, which is often identified as basal to *Saccharomycetaceae*, thus implying an origin in their last common ancestor. The three other examples demonstrate loss of genes in specific species, that are otherwise conserved across a clade, for example a set of 27 genes is lost in *Candida albicans* (which is a human infectious pathogen) but these are otherwise conserved across *Saccharomycetes*. Two clusters were picked out that lack orthologs in the representative *Pseudogymnoascus* species (a genus which includes a fatal bat pathogen (Isidoro-Ayza & Klein, 2024)), but are otherwise conserved across *Ascomycota* or *Fungi* generally, with some sporadic loss in other species in the *Ascomycota* example in part (D). These lacks are unlikely to be due to genome assembly quality, since the BUSCO C score is 99.2% for the *Pseudogymnoascus* species. The last example is for a cluster of profiles conserved across *Fungi*, but lacking in just *Pseudogymnoascus*, that is significantly linked to *transcription initiation at RNA polymerase II promoter* (GO:0006367, *P* = 9e−06) and also otherwise the cytoplasm compartment (GO:0005737, *P* = 7e−06) (The other examples have no significant enrichments or depletions of GO terms).

### Significant gene ontology associations for profile clusters

There is a total of 45 significant Gene Ontology (GO) functional term associations for profile clusters, most of which (26/45) are associated with gene origination in *Fungi* or earlier (Table S1). The most significant of these 26 is for a large cluster of 183 genes that have conserved absences in some non-*Ascomycota* species, linked to *amino acid biosynthetic process* (term identifier GO:0008652). A suite of significant associations for a large *Saccharomycetaceae*-linked cluster are linked to meiosis and cellular spore formation (terms GO:0051321, GO:0030435, GO:0007049, GO:0051301, GO:0007059). Cluster 3,958 comprising 10 genes and is labelled as most similar to the *S. cerevisiae* ideal profile, but has a low CD (0.88) and high CA (0.10), and demonstrates sporadic conservation across several diverse *Fungi*, and linkage to genes with functional roles in aerobic respiration (Table S1).

### Trends in intrinsically-disordered proteins, prion-like proteins and ‘unique’ yeast proteins

Proteins can be assigned to a certain evolutionary epoch, depending on the corresponding assignment of their profile clusters. We can vary this assignment using different CA and CD threshold levels, varying these in the range 0.8 ≤ CA ≤ 1.0, and 0.2 ≥ CD ≥ 0.0 (as described in Methods). In doing so, we can check for parameter independence of trends in the total population of proteins assigned to each epoch. Here, we are interested in the emergence of novel intrinsically-disordered or prion-like proteins for such populations. Also, we can discern in which eras ‘unique’ yeast proteins have emerged and subsequently become conserved.

**Figure 4:**
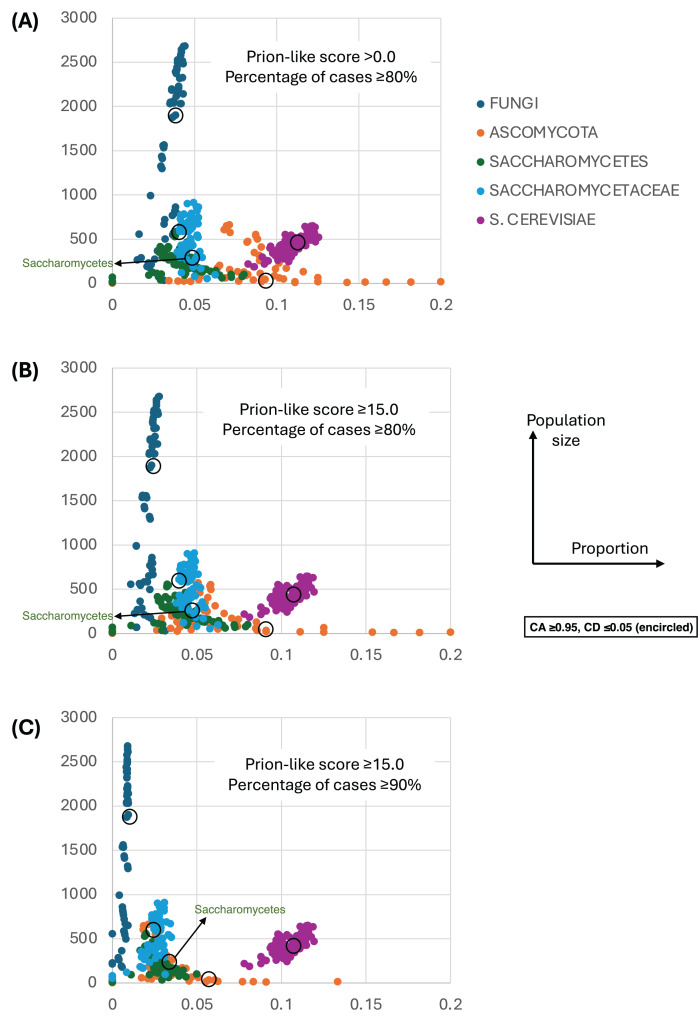
The proportions of prion-like proteins for different epochs of gene origination. (A) Each point is the total population of *S. cerevisiae* proteins whose profiles are assigned to a specific evolutionary epoch (from clusters of size ≥10 members). Size of population is plotted *vs* proportion that are prion-like with PLAAC program score >0.0 and percentage of cases across orthologs ≥80%. Each point is for a value of consensus agreement (CA) in the range 0.8–1.0 and consensus distance (CD) in the range 0.0–0.2, and for the *HQ1*, *HQ2* and *all* data sets. The epochs of gene origination are colour-coded for each cluster that has been assigned to that epoch according to the procedure described in *Methods*. As a ‘mid-range’ example, the data sets for CA ≥ 0.95 and CD ≤ 0.05 are encircled for each colour-coded ‘epoch’. (B) As in (A), but for score ≥15.0 and percentage ≥80%. (C) As in (A), but for score ≥15.0 and percentage ≥90%.

**Figure 5:**
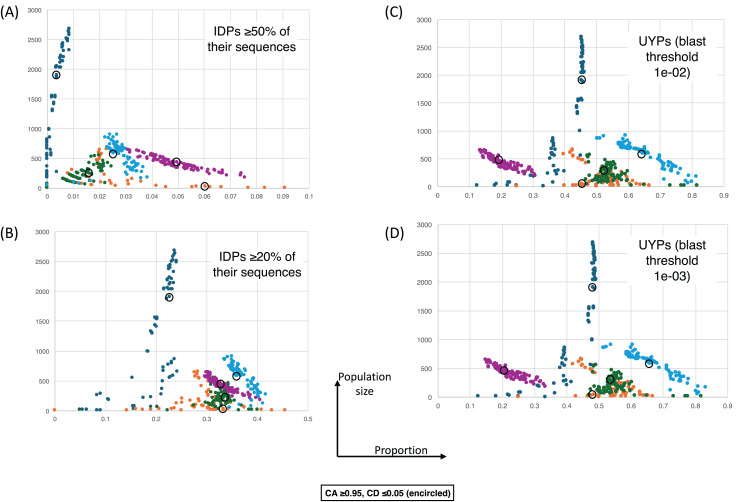
The proportions of intrinsically-disordered proteins and ‘unique’ yeast proteins (UYPs) for different epochs of gene origination. The colour coding is as for Fig. 4. Each point is for a value of CA in the range 0.8–1.0 and CD in the range 0.0–0.2, and for the *HQ1*, *HQ2* and *all* data sets. (A) As in Fig. 4, but for the size of population *vs* proportion of proteins that are intrinsically-disordered (IDPs) ≥50% of their sequences. CA and CD ranges for the points are the same. (B) As in (A), except for IDPs that are ≥20% intrinsically-disordered. (C) As in (A) except for the proportion of UYPs labelled using the 1e−02 BLASTP threshold. (D) As in (A) except for the proportion of UYPs labelled using the 1e-03 BLASTP threshold.

Figure 4 shows a largely parameter-independent trend in the emergence of proteins with conserved prion-like status. In this figure, the different panels are for different criteria for assigning prion-like proteins across an ortholog set, *e.g*., in Fig. 4A, the orthologs in a cluster must have a domain with a score >0.0 from the prion-like-domain finder program PLAAC, and these prion-like domains must occur across ≥80% of the orthologs of a cluster. In each panel, every point represents the total population of proteins assigned to each evolutionary epoch. For prion-like proteins with high PLAAC prion composition scores (≥15.0), it is clear that new prion-like proteins tend to emerge at a similar rate regardless of evolutionary epoch (~2–5% of new proteins), except for very recent new proteins (clustered at the *Saccharomyces* genus level), where the rate increases to ~10%. Similarly, new proteins that are intrinsically disordered over ≥50% of their sequences tend to arise more recently, particularly in the *Saccharomyces* genus (Figs. 5A and 5B). However, with a lower threshold (≥20% of sequences being intrinsically disordered), such proteins originating in *Saccharomycetaceae* become proportionally the most numerous, indicating a complex relationship between intrinsic disorder and the epoch of new gene origination (Figs. 5A and 5B). ‘Unique’ yeast proteins (UYPs) were defined in Methods. A clear trend in the emergence of new UYPs is observed, with notably fewer of these occurring particularly toward the *Saccharomyces* epoch, and those emerging in the *Saccharomycetaceae* era standing out as proportionally most enriched (Figs. 5C and 5D). This is also evident in the distributions of profile mismatches analyzed in Fig. 1, where this UYP data has been separated out.

## Discussion

Clustering of phylogenetic profiles offers an avenue to observe epochs in gene origination during evolution, without the application of arbitrary thresholds relating to the estimated gene origination point. Using this technique, we observed evidence for emergence of many new yeast genes in clear epochs, near the last common ancestor (LCA) of *Saccharomycetes*, *Saccharomycetaceae*, *Saccharomyces* genus and in *Fungi* and before, which form several clusters of phylogenetic profiles. Some of these have obvious functional associations, such as to cellular spore formation in the early evolution of *Saccharomycetaceae*, cellular spores being a survival state that can withstand heat and desiccation. Since the trends were also found when comparing to randomly-generated profiles, they are unlikely to be due to comparing against ‘ideal’ profiles for each of the epochs. The epochal trends are also confirmed when the individual profiles are clustered and their profile consensi evaluated.

One drawback of the profile clustering procedure is that for larger clusters it does not provide a clear estimation of the origination time of a set of genes during evolution. Also, any method based on standard sequence alignment will not sufficiently capture the evolution of proteins that have higher mutation rates over the whole of their sequences, but this is a general problem for any evolutionary analysis of such proteins using standard sequence alignment. For example, one type of protein that is difficult to align are proteins that are substantially intrinsically-disordered, however these are actually very rare (only ~4% of *S. cerevisiae* proteins have intrinsic disorder over greater than two thirds of their sequences). The existence of such proteins does not change the overall results with regards to specific peaks that correspond to distinct evolutionarily meaningful epochs during the ancestry of *S. cerevisiae*. A more complex alternative explanation for these peaks would be that large populations of older fast-evolving proteins are becoming slow evolving (and thus detectable through sequence alignment) at specific milestones back towards ancient fungal ancestry.

The profile cluster quality indicators (consensus distance, consensus agreement, and quality-level agreements) are significantly correlated indicating that a strong signal for concerted appearance of new genes near the LCA of key epochs is detectable regardless of the quality threshold applied to the proteome data. The implication is that there is a payoff between more data (which might still be correct) *vs* a greater chance of it being incorrect due to bad genome assembly.

Very few large profile clusters (≥10 members) were assignable as closest to the whole genome duplication *WGD* ideal profile (just two cases). It is possible that the lack of detection of such clusters is linked to variation in mutation rates. Previous analysis has indicated that when both ohnologs are conserved after the whole genome duplication event, one of them has very likely undergone a rapid mutation process subsequently (Kellis, Birren & Lander, 2004), which may confound detection of their concerted appearance. Profile clusters assigned to the *Ascomycota* LCA are in general more diffuse (having lower consensus agreement values, and higher consensus distance values), indicating more complex patterns of gene origination and loss, that might be resolved as more *Ascomycota* genomes continue to be sequenced and assembled.

It was previously discovered that there was a burst of formation of prion-like domains in *Saccharomycetes* proteomes, due primarily to the formation of tracts of poly-asparagine (An, Fitzpatrick & Harrison, 2016). Here however, we observed that the proportion of complete ‘new’ prion-like proteins does not differ much between the *Ascomycota, Saccharomycetes* or *Saccharomycetaceae* evolutionary levels (typically ~5%), and is in fact greatest (~10–12%) for very recently birthed genes. This implies that the new prion-like domains noted previously are mostly emerging in *existing* proteins through the new mutational biases that emerged in *Saccharomycetes* (An, Fitzpatrick & Harrison, 2016).

Highly-disordered new proteins (≥50% of their sequences) are a greater proportion of the new protein population, the more recently that they were formed. However, proteins that are at least moderately disordered (≥20%) are most numerous amongst new proteins that arose near the *Saccharomycetaceae* (*Sa*) LCA, and also a high proportion of these *Sa* new proteins (~60–80%) are ‘unique’ proteins with no alignable homologs within the yeast proteome. These trends may be linked to the specific functional enrichments observed (*i.e*., meiosis, chromosome segregation, cellular spore formation). Previous analyses with rather less proteome data (19 fungal proteomes) derived somewhat different results, and showed a peak in overall intrinsic disorder in new protein (or ‘orphan’ protein) populations at the *Saccharomycetes* level of origination (Ekman & Elofsson, 2010). The apparent ‘uniqueness’ of some of these proteins may simply also be due to rapid evolution in intrinsically-disordered proteins (Ekman & Elofsson, 2010); however such rapid evolution would also be expected to make such disordered proteins most numerous in proteins that arose in or close to the *Saccharomyces* genus. Surveys of other kingdoms indicate that such large-scale evolutionary trends in intrinsic disorder content may be clade-specific, with, for example, intrinsically-disordered plant protein domains showing an opposite correlation with their evolutionary age (James et al., 2021). Increased intrinsic disorder content may also be linked to the acquisition of greater cell-type diversity, as observed for centrosomal proteins across animals, with *S. cerevisiae* as a unicellular outgroup (Nido et al., 2012). Also, here, we have noted that it is important to treat sequences that have different levels of annotated intrinsic disorder differently, as they can have markedly various behaviour.

## Conclusions

It was shown that there is sufficient information in phylogenetic profiles to mine for gene origination trends. Significant functional associations and trends in prion-like composition, intrinsic disorder and gene ‘uniqueness’ were discovered. Phylogenetic profile clusters generated here (particularly those with very high values of cluster consensus agreement) might be useful for investigating experimental hypotheses, since they provide evidence for functional linkages that have yet to be discerned.

## Supplemental Information

10.7717/peerj.19370/supp-1 Supplemental Information 1 Distributions of profile mismatches (PM) *versus* two randomly-generated profiles. This distributions and labelling are the same as in Figure 1 except that in (A) the comparison is to a randomly-generated profile with a 0.25 chance of a 1 character, and in (B) with a 0.75 chance.

10.7717/peerj.19370/supp-2 Supplemental Information 2 Significant Gene Ontology (GO) term associations for profile clusters. Colour-coded for epochs as in the figures.

10.7717/peerj.19370/supp-3 Supplemental Information 3 Proteome list and quality data.

10.7717/peerj.19370/supp-4 Supplemental Information 4 Profile cluster information.

10.7717/peerj.19370/supp-5 Supplemental Information 5 Multiple profile alignments (MPAs) for clusters.

## Additional Information and Declarations

### Data Availability

The following information was supplied regarding data availability:

The list of proteomes and the profile cluster data are available in the Supplemental Files.

## References

[ref-1] Altschul SF, Madden TL, Schaffer AA, Zhang J, Zhang Z, Miller W, Lipman DJ (1997). Gapped BLAST and PSI-BLAST: a new generation of protein database search programs. Nucleic Acids Research.

[ref-2] An L, Fitzpatrick D, Harrison PM (2016). Emergence and evolution of yeast prion and prion-like proteins. BMC Evolutionary Biology.

[ref-3] Barrera-Redondo J, Lotharukpong JS, Drost HG, Coelho SM (2023). Uncovering gene-family founder events during major evolutionary transitions in animals, plants and fungi using GenEra. Genome Biology.

[ref-4] Carvunis AR, Rolland T, Wapinski I, Calderwood MA, Yildirim MA, Simonis N, Charloteaux B, Hidalgo CA, Barbette J, Santhanam B, Brar GA, Weissman JS, Regev A, Thierry-Mieg N, Cusick ME, Vidal M (2012). Proto-genes and de novo gene birth. Nature.

[ref-5] Domazet-Loso T, Brajkovic J, Tautz D (2007). A phylostratigraphy approach to uncover the genomic history of major adaptations in metazoan lineages. Trends in Genetics.

[ref-6] Ekman D, Elofsson A (2010). Identifying and quantifying orphan protein sequences in fungi. Journal of Molecular Biology.

[ref-7] Erdos G, Pajkos M, Dosztanyi Z (2021). IUPred3: prediction of protein disorder enhanced with unambiguous experimental annotation and visualization of evolutionary conservation. Nucleic Acids Research.

[ref-9] Grigoriev IV, Nikitin R, Haridas S, Kuo A, Ohm R, Otillar R, Riley R, Salamov A, Zhao X, Korzeniewski F, Smirnova T, Nordberg H, Dubchak I, Shabalov I (2014). MycoCosm portal: gearing up for 1000 fungal genomes. Nucleic Acids Research.

[ref-10] Hittinger CT, Rokas A, Bai FY, Boekhout T, Goncalves P, Jeffries TW, Kominek J, Lachance MA, Libkind D, Rosa CA, Sampaio JP, Kurtzman CP (2015). Genomics and the making of yeast biodiversity. Current Opinion in Genetics & Development.

[ref-11] Isidoro-Ayza M, Klein BS (2024). Pathogenic strategies of Pseudogymnoascus destructans during torpor and arousal of hibernating bats. Science.

[ref-12] James JE, Willis SM, Nelson PG, Weibel C, Kosinski LJ, Masel J (2021). Universal and taxon-specific trends in protein sequences as a function of age. eLife.

[ref-13] Kellis M, Birren BW, Lander ES (2004). Proof and evolutionary analysis of ancient genome duplication in the yeast Saccharomyces cerevisiae. Nature.

[ref-14] Lancaster AK, Nutter-Upham A, Lindquist S, King OD (2014). PLAAC: a web and command-line application to identify proteins with prion-like amino acid composition. Bioinformatics.

[ref-15] Manni M, Berkeley MR, Seppey M, Simao FA, Zdobnov EM (2021). BUSCO update: novel and streamlined workflows along with broader and deeper phylogenetic coverage for scoring of eukaryotic, prokaryotic, and viral genomes. Molecular Biology and Evolution.

[ref-16] Montanes JC, Huertas M, Messeguer X, Alba MM (2023). Evolutionary trajectories of new duplicated and putative de novo genes. Molecular Biology and Evolution.

[ref-17] Nido GS, Mendez R, Pascual-Garcia A, Abia D, Bastolla U (2012). Protein disorder in the centrosome correlates with complexity in cell types number. Molecular Bio Systems.

[ref-18] Pellegrini M (2012). Using phylogenetic profiles to predict functional relationships. Methods in Molecular Biology.

[ref-19] Pellegrini M, Marcotte EM, Thompson MJ, Eisenberg D, Yeates TO (1999). Assigning protein functions by comparative genome analysis: protein phylogenetic profiles. Proceedings of the National Academy of Sciences of the United States of America.

[ref-20] Roginski P, Grandchamp A, Quignot C, Lopes A (2024). De novo emerged gene search in eukaryotes with DENSE. Genome Biology and Evolution.

[ref-21] Saitou N, Nei M (1987). The neighbor-joining method: a new method for reconstructing phylogenetic trees. Molecular Biology and Evolution.

[ref-22] Su WC, Harrison PM (2020). Deep conservation of prion-like composition in the eukaryotic prion-former Pub1/Tia1 family and its relatives. PeerJ.

[ref-23] Aleksander SA, Balhoff J, Carbon S, Cherry JM, Drabkin HJ, Ebert D, Feuermann M, Gaudet P, Harris NL, Hill DP, Lee R, Mi H, Moxon S, Mungall CJ, Muruganugan A, Mushayahama T, Sternberg PW, Thomas PD, Van Auken K, Ramsey J, Siegele DA, Chisholm RL, Fey P, Aspromonte MC, Nugnes MV, Quaglia F, Tosatto S, Giglio M, Nadendla S, Antonazzo G, Attrill H, Dos Santos G, Marygold S, Strelets V, Tabone CJ, Thurmond J, Zhou P, Ahmed SH, Asanitthong P, Luna Buitrago D, Erdol MN, Gage MC, Ali Kadhum M, Li KYC, Long M, Michalak A, Pesala A, Pritazahra A, Saverimuttu SCC, Su R, Thurlow KE, Lovering RC, Logie C, Oliferenko S, Blake J, Christie K, Corbani L, Dolan ME, Drabkin HJ, Hill DP, Ni L, Sitnikov D, Smith C, Cuzick A, Seager J, Cooper L, Elser J, Jaiswal P, Gupta P, Jaiswal P, Naithani S, Lera-Ramirez M, Rutherford K, Wood V, De Pons JL, Dwinell MR, Hayman GT, Kaldunski ML, Kwitek AE, Laulederkind SJF, Tutaj MA, Vedi M, Wang SJ, D’Eustachio P, Aimo L, Axelsen K, Bridge A, Hyka-Nouspikel N, Morgat A, Aleksander SA, Cherry JM, Engel SR, Karra K, Miyasato SR, Nash RS, Skrzypek MS, Weng S, Wong ED, Bakker E, Berardini TZ, Reiser L, Auchincloss A, Axelsen K, Argoud-Puy G, Blatter MC, Boutet E, Breuza L, Bridge A, Casals-Casas C, Coudert E, Estreicher A, Livia Famiglietti M, Feuermann M, Gos A, Gruaz-Gumowski N, Hulo C, Hyka-Nouspikel N, Jungo F, Le Mercier P, Lieberherr D, Masson P, Morgat A, Pedruzzi I, Pourcel L, Poux S, Rivoire C, Sundaram S, Bateman A, Bowler-Barnett E, Bye AJH, Denny P, Ignatchenko A, Ishtiaq R, Lock A, Lussi Y, Magrane M, Martin MJ, Ruzicka L, Westerfield M, The Gene Ontology Consortium (2023). The Gene Ontology knowledgebase in 2023. Genetics.

[ref-24] The UniProt Consortium (2023). UniProt: the universal protein knowledgebase in 2023. Nucleic Acids Research.

[ref-25] Vakirlis N, Acar O, Cherupally V, Carvunis AR (2024). Ancestral sequence reconstruction as a tool to detect and study de novo gene emergence. Genome Biology and Evolution.

[ref-26] Wolfe KH, Shields DC (1997). Molecular evidence for an ancient duplication of the entire yeast genome. Nature.

