# Supplementary material for: Robust phylogenetic profile clustering for Saccharomyces cerevisiae proteins
Source: PeerJ. 2025 Apr 28;13:e19370. doi: 10.7717/peerj.19370 (PMC12045281; doi:10.7717/peerj.19370)

Suppl. Figure: Distributions of profile mismatches (PMs) versus two randomly-generated profiles

This distributions are the same as for the PM distribution figure in the main text, except that in (A) the comparison is to a randomly-generated profile with a 0.25 chance of a 1 character, and in (B) with a 0.75 chance. The labelling is as in Figure 1

(A) Random profile, 0.25 chance of 1, 0.75 chance of 0

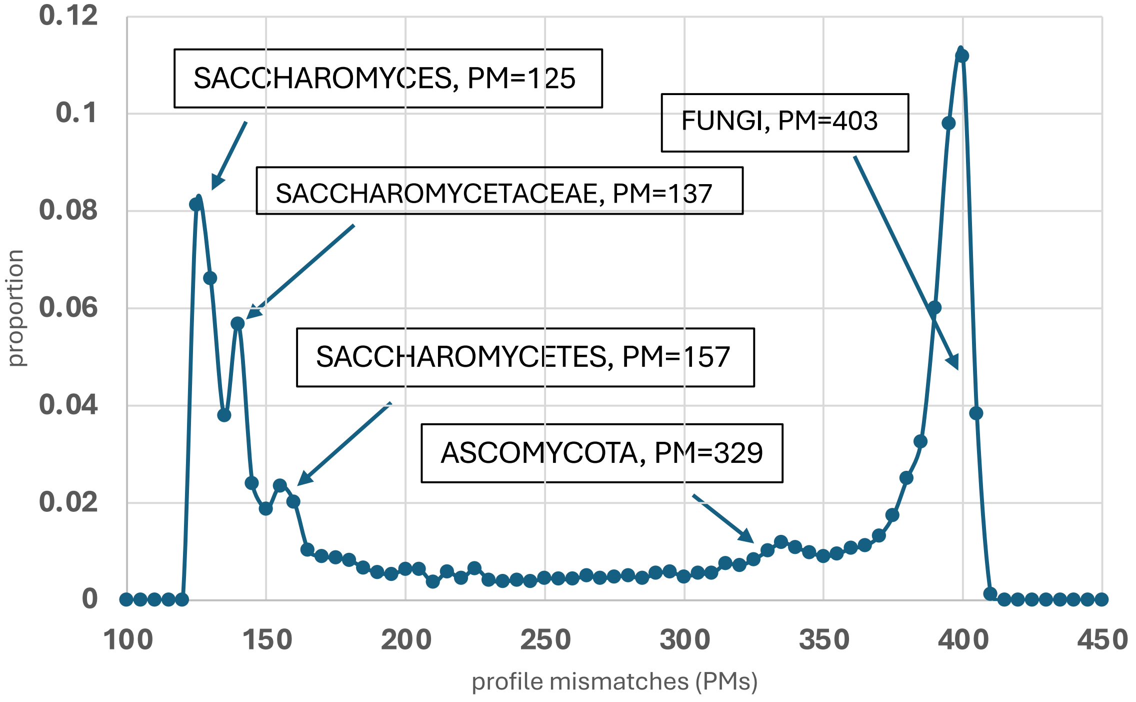

(B) Random profile, 0.75 chance of 1, 0.25 chance of 0

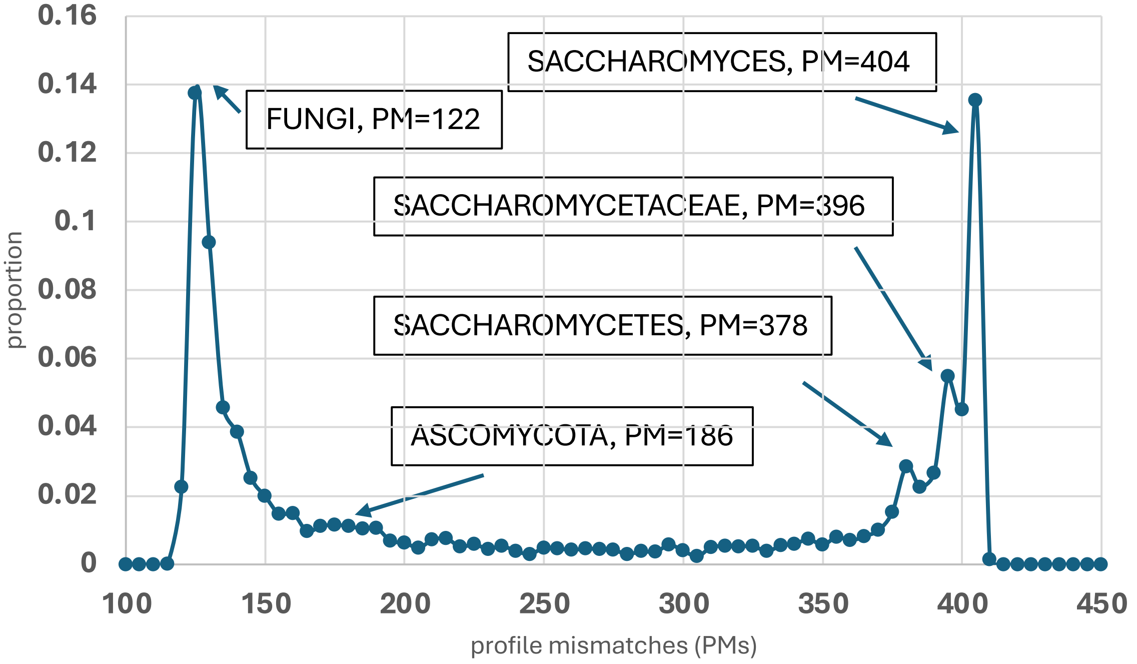

Supplement: Supplemental Information 1 — This distributions and labelling are the same as in Figure 1 except that in (A) the comparison is to a randomly-generated profile with a 0.25 chance of a 1 character, and in (B) with a 0.75 chance. [file peerj-13-19370-s001.pdf]
