# Supplementary material for: Robust phylogenetic profile clustering for Saccharomyces cerevisiae proteins
Source: PeerJ. 2025 Apr 28;13:e19370. doi: 10.7717/peerj.19370 (PMC12045281; doi:10.7717/peerj.19370)
Supplement: Supplemental Information 2 — Colour-coded for epochs as in the figures. [file peerj-13-19370-s002.docx]

| **Cluster ID**  **Supplementary Table: Significant Gene Ontology (GO) term associations for profile clusters*** | **Epoch** | **GO term** | **Description** | **Cluster Size** | **GO term cluster count** | **GO term proteome count** | **P-value** |
| --- | --- | --- | --- | --- | --- | --- | --- |
| **5272** | **Sa** | **GO:0051321** | **Meiotic cell cycle** | **627** | **42** | **128** | **5.3e-12** |
| 5176 | F | GO:0008652 | Amino acid biosynthetic process | 183 | 17 | 88 | 9.2e-10 |
| **5249** | **A** | **GO:0003700** | **DNA-binding TF activity** | **550** | **23** | **60** | **1.0e-09** |
| 3917 | F | GO:0006487 | Protein N-linked glycosylation | 10 | 5 | 34 | 1.2e-09 |
| 5140 | F | GO:0016491 | Oxidoreductase activity | 144 | 25 | 271 | 4.9e-09 |
| 5140 | F | GO:0034354 | ‘de novo’ NAD biosynthetic process from tryptophan | 144 | 5 | 6 | 4.9e-08 |
| 4270 | F | GO:0042719 | Mitochondrial intermembrane space protein transporter complex | 16 | 3 | 4 | 6.6e-08 |
| 4794 | F | GO:0000166 | Nucleotide binding | 52 | 22 | 757 | 9.7e-08 |
| **3958** | **Sc** | **GO:1902600** | **Proton transmembrane transport** | **10** | **5** | **90** | **1.8e-07** |
| 4270 | F | GO:0140318 | Protein transporter activity | 16 | 3 | 6 | 3.3e-07 |
| 4270 | F | GO:0005758 | Mitochondrial intermembrane space | 16 | 5 | 62 | 4.4e-07 |
| **5249** | **A** | **GO:0000981** | **DNA-binding TF activity, RNA pol.-II specific** | **550** | **29** | **118** | **5.6e-07** |
| 5184 | F | GO:0000386 | Second spliceosomal transesterification activity | 229 | 6 | 10 | 5.9e-07 |
| **3958** | **Sc** | **GO:0009060** | **Aerobic respiration** | **10** | **4** | **46** | **6.6e-07** |
| 4328 | F | GO:0000794 | Condensed nuclear chromosome | 17 | 4 | 26 | 6.8e-07 |
| **3958** | **Sc** | **GO:0006123** | **Mitochondrial electron transport, cyt. c to O_2_** | **10** | **3** | **13** | **1.0e-06** |
| 4794 | F | GO:1990825 | Seq.-specific mRNA binding | 52 | 5 | 22 | 1.0e-06 |
| 4629 | F | GO:0006487 | Protein N-linked glycosylation | 34 | 5 | 34 | 1.2e-06 |
| **5272** | **Sa** | **GO:0030435** | **Sporulation resulting in formation of cellular spore** | **627** | **28** | **103** | **1.4e-06** |
| 4740 | F | GO:0005634 | Nucleus | 44 | 32 | 2207 | 1.7e-06 |
| 3917 | F | GO:0006486 | Protein glycosylation | 10 | 4 | 60 | 2.0e-06 |
| **3958** | **Sc** | **GO:0004129** | **Cytochrome c oxidase activity** | **10** | **3** | **16** | **2.0e-06** |
| **3958** | **Sc** | **GO:0070469** | **Obsolete respirasome** | **10** | **3** | **16** | **2.0e-06** |
| **3958** | **Sc** | **GO:0005751** | **Obsolete mitochondrial resp. chain complex IV** | **10** | **3** | **16** | **2.0e-06** |
| **5272** | **Sa** | **GO:0007049** | **Cell cycle** | **627** | **58** | **303** | **2.5e-06** |
| 4123 | F | GO:0032258 | Cytoplasm to vacuole targeting by the Cvt pathway | 13 | 4 | 48 | 2.6e-06 |
| 4270 | F | GO:0045039 | Protein insertion into mitochondrial inner membrane | 16 | 3 | 11 | 2.7e-06 |
| **5272** | **Sa** | **GO:0051301** | **Cell division** | **627** | **42** | **194** | **2.7e-06** |
| **5065** | **Sc** | **GO:0071944** | **Cell periphery** | **104** | **17** | **268** | **2.9e-06** |
| 4450 | F | GO:0019509 | L-methionine salvage | 22 | 3 | 9 | 3.8e-06 |
| 4655 | F | GO:0000166 | Nucleotide binding | 37 | 16 | 757 | 3.8e-06 |
| 4794 | F | GO:0005524 | ATP binding | 52 | 18 | 652 | 4.6e-06 |
| **4032** | **Sc** | **GO:0043328** | **Protein transport to vacuole in ubiquitin-dept. protein catabolic process** | **12** | **3** | **18** | **5.2e-06** |
| 5140 | F | GO:0009435 | NAD biosynthetic process | 144 | 5 | 12 | 5.5e-06 |
| 4328 | F | GO:0007129 | Homologous chromosome pairing at meiosis | 17 | 3 | 13 | 5.6e-06 |
| **5272** | **Sa** | **GO:0007059** | **Chromosome segregation** | **627** | **22** | **76** | **5.9e-06** |
| **5065** | **Sc** | **GO:0003993** | **Acid phosphatase activity** | **104** | **4** | **8** | **6.1e-06** |
| **4673** | **Sc** | **GO:0000981** | **DNA-binding TF activity, RNA pol.-II specific** | **37** | **7** | **118** | **6.4e-06** |
| 5176 | F | GO:0005759 | Mitochondrial matrix | 183 | 15 | 128 | 6.8e-06 |
| 4934 | F | GO:0005737 | Cytoplasm | 76 | 50 | 2412 | 7.0e-06 |
| **5249** | **A** | **GO:1902047** | **Polyamine transmembrane transport** | **550** | **5** | **5** | **7.1e-06** |
| 3917 | F | GO:0016757 | Glycosyltransferase activity | 10 | 4 | 83 | 7.2e-06 |
| **5249** | **A** | **GO:0043565** | **Seq.-specific DNA binding** | **550** | **26** | **113** | **7.4e-06** |
| 4740 | F | GO:0008033 | tRNA processing | 44 | 7 | 101 | 7.6e-06 |
| 4934 | F | GO:0006367 | Transcription initiation at RNA pol. II promoter | 76 | 5 | 23 | 8.6e-06 |

* colour-coded for epochs in the same way as in the figures.
